# Biomechanical Reconstructions and Selective Advantages of Neck Poses and Feeding Strategies of Sauropods with the Example of *Mamenchisaurus youngi*

**DOI:** 10.1371/journal.pone.0071172

**Published:** 2013-10-30

**Authors:** Andreas Christian, Guangzhao Peng, Toru Sekiya, Yong Ye, Marco G. Wulf, Thorsten Steuer

**Affiliations:** 1 Abteilung für Biologie und ihre Didaktik, Universität Flensburg, Flensburg, Germany; 2 Zigong Dinosaur Museum, Dashanpu, Zigong City, Sichuan Province, China; Raymond M. Alf Museum of Paleontology, United States of America

## Abstract

A very long neck is a characteristic feature of most sauropod dinosaurs. In the genus *Mamenchisaurus*, neck length is extreme, greater than 40 percent of total body length. However, the posture, utilization, and selective advantage of very long necks in sauropods are still controversial. An excellently preserved skeleton of *Mamenchisaurus youngi*, including a complete neck, provides an opportunity for a comprehensive biomechanical analysis of neck posture and mobility. The biomechanical evidence indicates that *Mamenchisaurus youngi* had a nearly straight, near horizontal neck posture and browsed at low or medium heights. The results differ from the findings for some other sauropod species, like *Euhelopus*, *Diplodocus*, and *Giraffatitan* (*Brachiosaurus*) that had been analyzed in previous studies with similar methods. The selective advantage of extreme neck length in sauropods is likely advantageous for different feeding strategies.

## Introduction

The very long neck is a characteristic feature of most sauropods [1], [2] and possibly a key innovation for sauropod gigantism [1], though shorter necks occur in some species [3]. The implications of having a long neck have been intensively discussed, not only for sauropods but other extinct and living vertebrates as well [4]. According to recent findings, sauropods grew fast and consequently had a high metabolic rate (e.g., [1], [5]). Therefore, the rate of food intake must have been very high, and access to substantial food resources would have been essential.

Long necks appear to be obviously beneficial for high browsing because sauropods would have had access to food resources other herbivores could not reach (e.g., [6], [7]). However, the question whether some sauropods like *Giraffatitan* (formerly *Brachiosaurus*) *brancai* [8]–[10] actually browsed at great heights with a steeply inclined neck remains controversial [11], [12]. For other genera like *Diplodocus*, *Apatosaurus* [13], and *Nigersaurus* [14] most researchers agree on a low browsing strategy, though high browsing in a bipedal or tripedal stance appears possible for some sauropods that are usually regarded as low-browsers like *Diplodocus* [15]. Similar to high browsing, low browsing with a long neck might have been useful for reaching otherwise difficult or impossible to exploit resources, e.g., at shorelines or in swampy environments [12], [13].

The major selective advantage of a long neck might have been a reduction in energy costs because less energy was needed to move the long but lightly built neck than the very large, massive body (e.g., [1], [16], [17]). Depending on the distribution of food, this argument holds true for browsing at great heights [18] as well as for browsing at medium or low heights [19], even if high browsing evoked a very high blood pressure (see e.g., [18] versus [20]). In this study browsing height is classified relative to the dimensions of the sauropod instead of using absolute values. The term low browsing is used for feeding with the head below the height of the shoulders, or more precisely, with the head below the height of the vertebral centra at the neck-trunk transition, so that the neck is in a declining position. There is no clear separation between medium and great heights. However, with medium heights we classify here browsing with the head kept between shoulder level and a half neck length above the shoulders which means a neck inclination of about 30 degrees. Browsing with a neck that is inclined by more than 30 degrees is classified as high browsing.

Another advantage of a very long neck could have been a reduction in the time intervals between feedings, thus a higher percentage of active time of a sauropod could have been used for feeding (see discussion). Explanations for the extreme neck length of sauropods different from feeding advantages, e.g. sexual selection or thermoregulation, appear unlikely [4], [21].

Among terrestrial vertebrates, very long necks are not common. Because of the success of sauropods and the rare exceptions of shorter necks among this group of dinosaurs, it appears reasonable to assume that the selective advantage of a very long neck was enhanced by other characteristic sauropod features such as the bird-like respiratory system with air sacs in the neck, which reduced neck weight without reducing lever arms of neck muscles, tendons and ligaments; the absence of mastication, which meant the skull could remain small; and the high metabolic rate for which a high rate of food intake was necessary [1]. Very long necks were not restricted to sauropods of very large size, but are also common among much smaller species, like *Europasaurus* [22], as well. Therefore, the selective advantage of a long neck was not firmly correlated with very large body size [4].

Extreme neck length, even in comparison to other sauropods, is a characteristic feature of mamenchisaurids [23]–[25]. This study focuses on *Mamenchisaurus youngi* with a neck length of about 41% of the total body length [23], [24]. The skeleton of specimen ZDM0083 is excellently preserved, including a complete neck and head [23], [24], making *Mamenchisaurus youngi* an ideal example for studying the neck mechanics and the feeding strategy of a sauropod with an extremely long neck. The neck skeleton was analyzed in order to reconstruct its posture and mobility. Based on the results of the biomechanical analysis, possible feeding strategies are discussed for *Mamenchisaurus youngi*.

## Materials and Methods

### Materials

Measurements were taken from the skeletal remains of *Mamenchisaurus youngi*, specimen ZDM0083 of the Zigong Dinosaur Museum, Zigong, Sichuan, China [23]. Additional data were taken from the description and the illustrations by Ouyang and Ye [24]. Data lacking due to damaged vertebrae were interpolated.

### Osteologically Neutral Pose (ONP) and neck mobility

The ONP of the neck is the zygapophyseal alignment posture. The ONP was determined by bringing the post- and prezygapophyses of adjacent vertebrae into contact, so that the joint between the centra was articulated and the joint facets of the pre- and postzygapophyses were centered above each other.

For this analysis, depending on their shapes, cotyles were placed into the adjacent condyles so that a close and smooth fit between both surfaces was obtained. The layer of cartilage between cotyles and condyles of adjacent vertebrae was assumed to be thin, between one or two centimeters on average for most parts of the neck and even less in the foremost region of the neck. Depending on the shape of cotyles and condyles the cartilage might have been thicker at some midpoints of the intervertebral joints; this, however, would not have affected the analysis. The assumption of rather thin layers of cartilage between the vertebral centra was derived from the usually close fit of cotyles and condyles. The neck of *Mamenchisaurus youngi* was preserved in articulation [23]. Although some vertebrae were separated after death, others were still found in close contact. A large fraction of the cotyle of the fifteenth cervical is still sitting deeply in the condyle of the sixteenth cervical, leaving not much space for cartilage. Several articulated neck vertebrae of related species can be found in situ in the bone beds of Zigong. The close and tight fit of these vertebrae corroborates the assumption of a rather thin layer of cartilage between cotyles and condyles. Therefore, the possible error in the estimated angulations of adjacent vertebrae due to uncertainties in the estimates of the thickness of joint cartilage is not more than two or three degrees.

Maximum dorsal mobility was estimated by tilting articulated vertebrae dorsally until the bone stopped further movement. Ventral and lateral flexibility are more difficult to estimate [26]. For ventral flexibility, it was assumed that the articulating zygapophyseal surfaces did not completely lose contact [26]. Lateral flexibility was only roughly estimated by the size of the zygapophyseal joint surfaces. The dorsoventral mobility of adjacent vertebrae was tested directly by bringing articulated vertebrae into the extreme positions described above, or, if this was not possible, e.g., due to deformations of the vertebrae, maximum excursions at the intervertebral joints were tested with the help of photographs taken of the vertebrae in side-view.

The surface area of the joint facets of the zygapophyses was estimated by assuming an elliptical shape. For the calculation of a surface area, its length and width were used as major axes of the ellipse. Of the two zygapophyseal joints between adjacent vertebrae, the best preserved joint facet was used for the estimates. The data are presented in Table S1.

### Stress in the intervertebral cartilage

Based on the dimensions of the neck skeleton, the volume of each neck segment was estimated, assuming that the dorsoventral outlines of the neck closely fit the reconstruction of the neck skeleton given in Plate II in [24]. An elliptical shape was assumed for most parts of the neck, with the transversal diameter being three quarters of the dorsoventral diameter. From the first to the fourth cervical vertebrae, additional mass was added for extra muscles that were needed for neck movements (e.g., [18], [26]–[28]). From the 15^th^ to the 18^th^ cervical vertebrae, a transition towards a round cross-section was assumed because of the considerable increase in the transversal diameter of the cervicals starting around the 15^th^ neck vertebra (for the basic data see Table S2). Mass distribution along the neck was reconstructed under the assumption of a very low neck density (0.5 gcm^−3^) due to large air volumes, generally suggested for sauropods by recent research (e.g., [29]–[31]). The mass of the head was approximated by assuming an ellipsoid fit closely around the head skeleton and a density of 0.9 gcm^−3^. A sensitivity analysis was conducted by using a horizontal neck posture and varying neck density between 0.4 gcm^−3^ and 0.7 gcm^−3^. Additionally, for a horizontal position of the neck with a density of 0.5 gcm^−3^, the mass of the head and the foremost section of the neck were varied. Also, a calculation was conducted with a very light base of the neck in order to demonstrate that the method is very robust against errors in mass estimates for the caudal section of the neck.

For different hypothetical neck postures, the stress in the intervertebral cartilage was calculated along the neck (Preuschoft-method; for a detailed description see [18], [27], [28], [32], [33]). The Preuschoft-method is based on the assumption of equal stress in the intervertebral cartilage along the neck in habitual neck postures [33]. This assumption is a consequence of Wolff's law [34] applied to cartilage. According to Wolff's law, bone adapts to loads. Bone is added where stress is high and removed where stress is low, so that under typical loading conditions stress is more or less constant throughout the bone, as has been corroborated in several recent studies (e.g., [35]). This concept was applied to intervertebral cartilage by Preuschoft [36] in order to reconstruct the spatial orientation of a vertebral column. The assumption of mean average stress in the intervertebral cartilage along the vertebral column was successfully tested for several terrestrial vertebrates [32], [33]. For camels and giraffes it was shown that the Preuschoft-method is a robust and reliable instrument for the reconstruction of the habitual neck posture of long-necked terrestrial vertebrates [33].

For sauropods, stress in the intervertebral cartilage is mainly due to bending moments along the neck. Theses bending moments are counteracted at the intervertebral junctions by tensile forces in epaxial muscles, tendons, or ligaments [32], [33], [36]. The tensile force of the epaxial muscles, tendons and ligaments produces a compressive force of the same magnitude that acts on the cartilage in the intervertebral joint in addition to gravity [32], [33], [36]. Thus, knowing the cross-sectional area of an intervertebral joint, the stress acting in the cartilage can be calculated [32], [33], [36], [37].

The lever arms of the epaxial forces were estimated by the vertical distances between the centers of the intervertebral joints and the tips of the neural spines [32], [33]. The cross-sectional area of the intervertebral joints is calculated by assuming an elliptical shape of the joints, with the transversal and dorsoventral diameters of the cranial surface of the adjacent vertebral centrum used as the major axes [32], [33]. Forces different from static or quasistatic forces are neglected, assuming that forces due to accelerations or other activities are negligible, except in the foremost region of the neck, where forces for positioning and accelerating the head cannot be excluded [27], [32], [33]. A hypothetical posture of the neck is rejected if the stress is not approximately constant along the neck. The basic data for the calculations of stress values are presented in Tables S2, S3, S4.

In order to compare the variation of stress values along the neck, mean stress (MS) and standard deviation (SD) of stress values divided by mean stress in the intervertebral cartilage (SD/MS) are calculated for the intervertebral joints along the neck for all hypothetical neck postures, starting at the intervertebral joint between the fifth and sixth vertebrae (c5–c6) and ending at the joint behind the fourteenth vertebra. The foremost section of the neck is not included in the calculations because the stress values in the foremost section of the neck are biased by additional forces for moving and positioning the head. The caudal section of the neck was not included because of a probable bias due to muscles and ligaments that might were located well above the neural spines [32], [33]. The higher SD/MS is the lower is the probability of the neck reconstruction.

If a sauropod frequently used different neck postures, the Preuschoft-method reveals the posture that evokes the highest stress along the neck. In the case that the stress curves of different frequently used neck postures intersect, the situation becomes complicated because the dimensions of the intervertebral discs in different sections of the neck might be determined by different neck poses.

## Results

### ONP and neck flexibility

The optimal fit of the pre- and postzygapophyseal joint surfaces yields a nearly straight neck posture with a slight upward bend at the base of the neck and a slight downward bend close to the head. Assuming the vertebral column of the trunk was slightly declining towards the shoulders because of the greater length of the hindlimbs compared to the forelimbs [24], the neck was kept close to the horizontal with an upward inclination of about 20 degrees.

**Figure 1:**
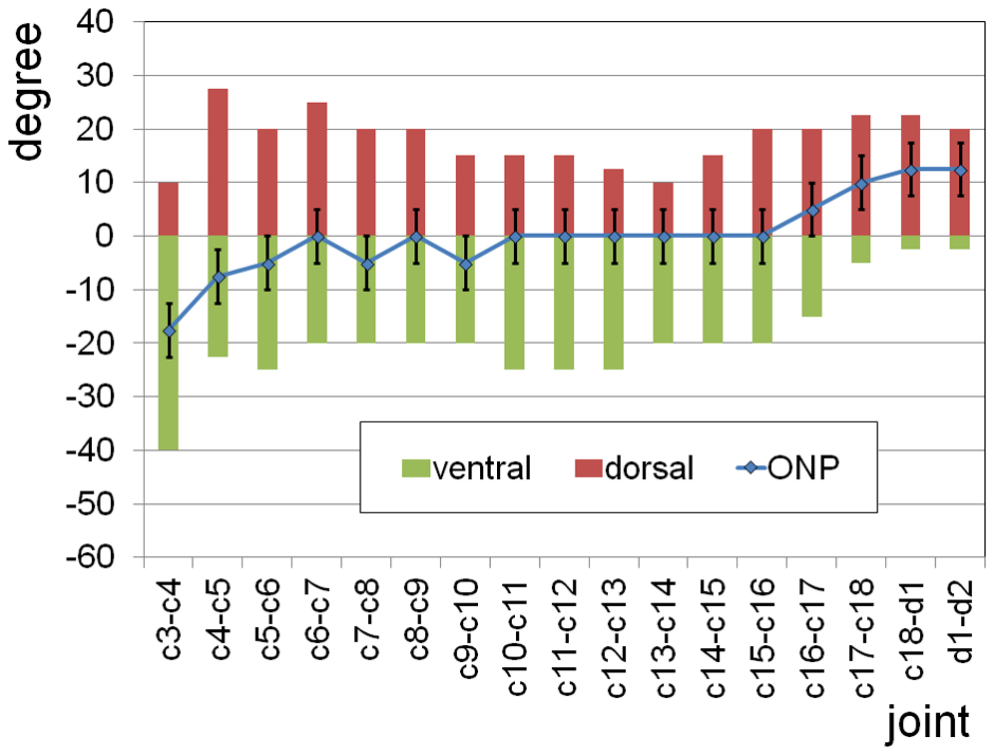
Osteologically Neutral Pose (ONP) and maximum dorsoventral excursions at the intervertebral joints along the neck and at the neck-trunk transition of *Mamenchisaurus youngi*. The angles are relative to a straight line of the middle axes of the vertebral centra. Positive angles mean dorsiflexion. For most joints in the midsection of the neck the ONP is straight. *c1*–*c18*, cervical vertebrae, *d1*,*d2*, first two dorsal vertebrae. An error of up to 5 degrees has to be taken into account for all angles due to deformations of the vertebrae and uncertainties in the estimate of the thickness of the intervertebral cartilage.

Estimates of maximum dorsoventral flexion at the intervertebral joints of the neck and the foremost section of the trunk are presented in Figure 1 and Table S5. According to results on living vertebrates with long necks, the estimated limits for dorsal flexion by bone-bone contact of adjacent vertebrae appear to be close to the excursion that can occur during daily activities. However, such extreme excursions do not occur frequently. For *Mamenchisaurus*, excursions close to bone contact are only likely at the neck-trunk transition, where the vertebral bone appears to form broad contact areas that prevented peak forces during extreme dorsal flexion. Therefore, the values presented in Figure 1 are extremes that were possibly reached rarely if at all. Ventral flexion might have not usually exceeded about two thirds of the values given in Figure 1, so that an overlap of one third of the joint surfaces in the zygapophyses was maintained. For ventral flexibility, extreme values probably were restricted to short sections of the neck. In the case that a long ligament extended above the tips of the neural spines, as it was observed in extant vertebrates with long necks [26], maximum flexion was restricted if long sections of the neck were involved. The dorsoventral flexibility is much lower if a minimum overlap of the zygapophyseal joint facets of 50% is assumed [13]. This assumption, however, appears not justified in the light of the results on extant vertebrates with long necks [26].

Despite the problems in defining the actual limits in dorsoventral excursions at the intervertebral joints, the results allow for some basic conclusions to be made on neck mobility in *Mamenchisaurus youngi*. Dorsoventral flexibility of the vertebral column of the neck decreases from head to trunk, similar to the ostrich [26], but less pronounced. Data are missing for the joint between the second and the third cervical vertebrae because the zygapophyses were not sufficiently preserved. The high ventral and low dorsal flexibility between the third and the fourth cervical indicate a predominance of downward movements in the foremost section of the neck. Dorsal flexibility reaches a maximum but decreases towards the midsection of the neck, where ventral flexibility is high. Further posterior, dorsal flexibility increases and ventral flexibility decreases. At the neck-trunk transition dorsal flexibility is comparatively high whereas ventral flexibility is very low.

**Figure 2:**
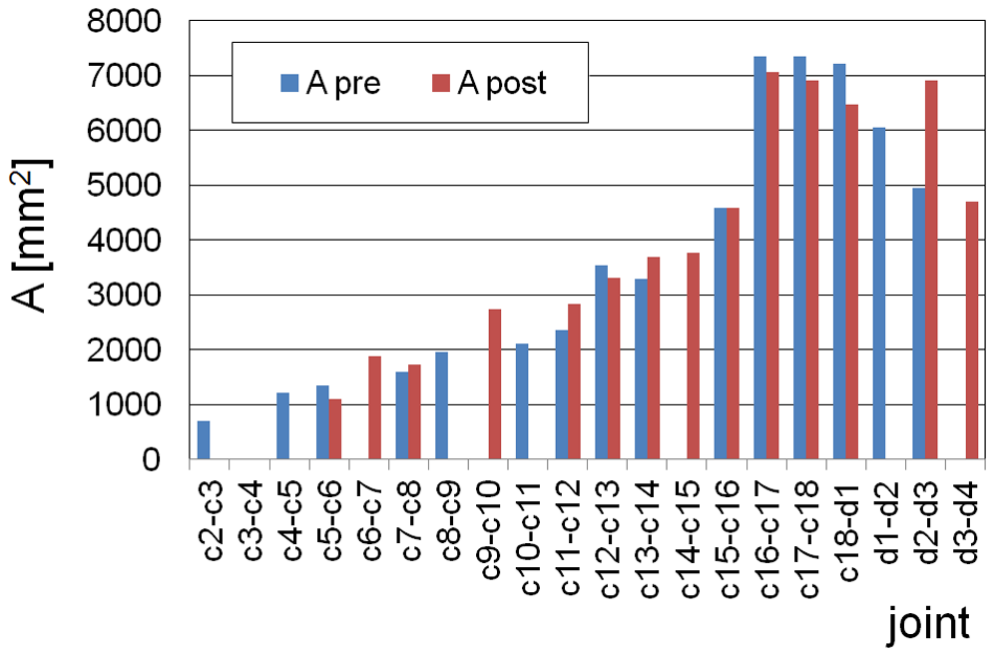
The size of the zygapophyseal joint facets. The surface area A of the joint facets is estimated for the prezygapophyses (A pre) and for the postzygapophyses (A post) by assuming an elliptical shape. Of both zygapophyseal joints between adjacent vertebrae, the best preserved joint facet is used for the estimates. In case of slight deformations or other damages, the joint surface was reconstructed, and in case of severe damage, no data are given. The estimated error due to deformation and deviation from elliptical shape of the joint facets is about ten percent.

Lateral flexibility of the neck is more difficult to derive from the skeleton alone [26]. However, the size and the shape of the zygapophyses provide some hints about the general pattern [13], [26], [38]. Between the second and third cervical vertebrae, the zygapophyseal joint facet is broad and compared to the length of the vertebrae rather large. Behind the fourth vertebra, the zygapophyseal joints are more or less of elliptical shape with the long axis approximately parallel to the neck and comparatively small. The joint facets are medially inclined by roughly 45 degrees. Starting at around the 15^th^ cervical, the vertebrae become much wider thereby increasing the lateral distance between the zygapophyses on both sides of the vertebrae. Simultaneously, the joint facets of the zygapophyses become much larger (Figure 2, Table S1), especially in width, so that starting around the 14^th^ cervical, the orientation of the long axis of the zygapophyseal joint facets is more lateral than longitudinal, and towards the neck-trunk transition, the inclination of the zygapophyseal joint facets is reduced. These findings indicate that lateral mobility is low in most parts of the neck, except the foremost section, but considerably increases towards the neck-trunk transition.

### Neck mass and stress in the intervertebral joint cartilage

The estimated combined mass of neck and head of *Mamenchisaurus youngi* is approximately 391 kg. With a straight neck, the distance between the snout and the base of the neck is estimated at 6.47 m. The data on neck segment length and mass used for the mechanical calculations are given in Table S2 and Table S3. The data on lever arms and cross-sectional areas of the intervertebral joints are presented in Table S4. Neck and head mass estimates for different neck shapes and densities are presented in Table 1 and Table S3.

**Table 1:** Stress values along the neck of *Mamenchisaurus youngi* for different neck reconstructions.

Reconstruction	d [gcm^−3^]	m [kg]	MS	SD/MS
horizontal	0.5	391.25	0.793	0.040
inclined sigmoid	0.5	391.25	0.640	0.134
declined (−15^0^)	0.5	391.25	0.645	0.040
inclined 45^0^	0.5	391.25	0.741	0.051
inclined 60^0^	0.5	391.25	0.506	0.069
horizontal d 0.4	0.4	318.00	0.708	0.061
horizontal d 0.6	0.6	464.50	0.877	0.041
horizontal d 0.7	0.7	537.75	0.962	0.055
heavy head (horizontal)	0.5	398.20	0.890	0.060
light head (horizontal)	0.5	384.30	0.696	0.049
light neck base (horizontal)	0.5	357.99	0.793	0.040

**Figure 3:**
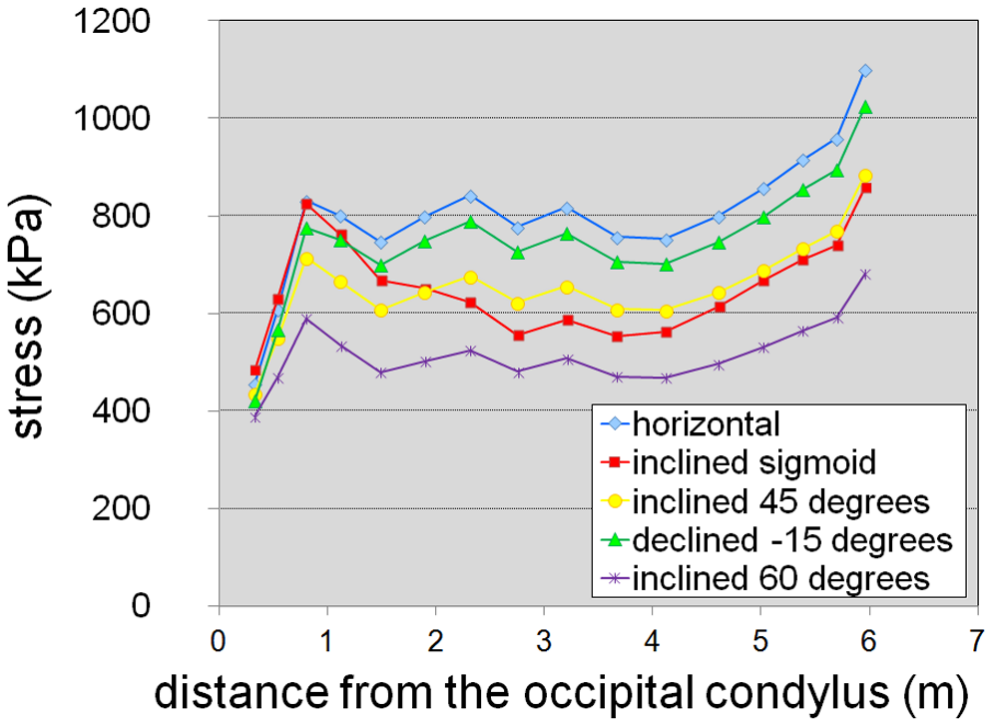
Stress in the intervertebral cartilage along the neck for different hypothetical neck postures (four straight postures and a sigmoid posture [[24], Plate II]).

Mean stress (MS) and standard deviation (SD) divided by mean stress in the intervertebral cartilage along the neck for the different neck reconstructions in Figures 3–5, starting at the intervertebral joint between the fifth and sixth vertebrae (c5–c6) and ending at the joint behind the fourteenth vertebra. The higher SD/MS is the lower is the probability of the neck reconstruction. For further explanation see the text. Estimated head mass is 25 kg, except for the “heavy head” and the “light head” reconstructions. In the “heavy head” reconstruction head mass is 30 kg and the mass of the foremost neck section between the first and the third cervical vertebrae is also increased by 20% (approximately 2 kg). In the “light head” reconstruction head mass is 20 kg and the mass of the foremost neck section between the first and the third cervical vertebrae is reduced by 20% (approximately 2 kg). In the “light neck base” reconstruction, the shape of the neck is maintained elliptical at its base instead of becoming circular towards the end. Segment mass estimates are presented in Table S3, stress values are given in Table S6. d, assumed density of the neck; m, combined mass of neck and head.

**Figure 4 pone-0071172-g004:**
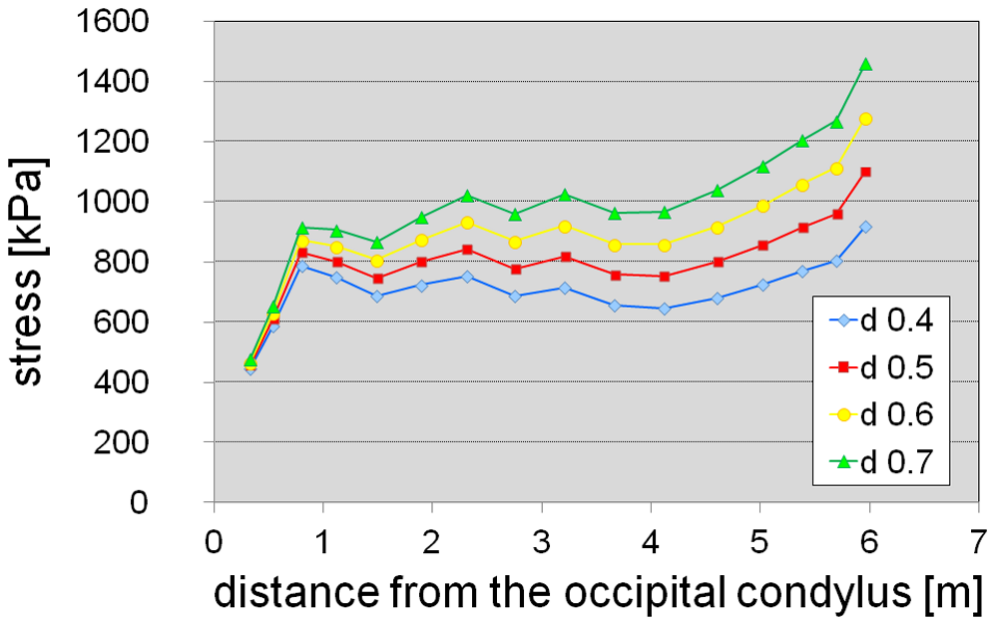
Stress in the intervertebral cartilage along the neck for different neck densities. The neck was assumed to be in a horizontal position. d 0.4–d 0.7, neck reconstructions assuming a density between 0.4 gcm^−3^ and 0.7 gcm^−3^.

For some hypothetical neck postures, the calculated stresses in the intervertebral joints along the neck are presented in Figure 3 and Table S6; average values and standard deviations divided by mean values are given in Table 1. The magnitude of the stress values is similar to estimates for other sauropods [18], [27] as well as our own estimates for some living vertebrates, and is also in accordance to the results of in vivo measurements of the pressure in an intervertebral disc of a human, which were 0.5 MPa for relaxed standing and 1.1. MPa for standing flexed forward [39]. Therefore, the overall mass estimate for the neck appears reasonable. A variation of neck density between 0.4 gcm^−3^ and 0.6 gcm^−3^, which is equivalent to a variation of neck mass by 20%, yields reasonable results for stress (Figure 4, Table 1). With a neck density of 0.7 gcm^−3^, which is equivalent to a 40% higher estimate of neck mass, stress values in the cartilage along the neck are about 1 MPa in a horizontal position and appear rather high for a relaxed pose of the neck. This indicates that even higher mass estimates for the neck of *Mamenchisaurus youngi* do not appear reasonable.

Nearly constant stress values in the intervertebral cartilage along the neck were obtained in straight neck poses for a slightly declined neck up to an inclination of the neck of about 45 degrees (Figure 3, Table 1). Because of uncertainties in the estimates of head and neck segment masses, habitual neck postures inside this range of inclinations are possible. Considerably bended neck postures (e.g., [24], Plate II) do not fit the expectation of constant stress in the intervertebral cartilage along the neck. These results indicate that the neck was generally kept in a more or less straight pose, with possible exceptions at both ends, close behind the head and in the region of the neck-trunk transition.

Very low stress close behind the head and high stress at the neck-trunk transition are observed in all poses tested for *Mamenchisaurus youngi*. These stress levels are seen in other sauropods as well (e.g., [18], [27], [33]). The low values observed close behind the head indicate additional forces due to head movements [18], [27]. The high values at the posterior end of the neck indicate that the lever arms of epaxial forces are underestimated. Presumably neck muscles, tendons or ligaments that connected the trunk with the neck were located well above the neural spines at the base of the neck as suggested for other sauropods [18], [27], [28], [33].

**Figure 5:**
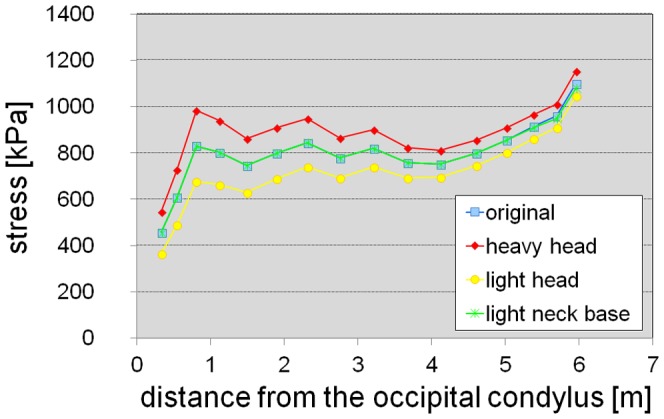
Stress in the intervertebral cartilage along the neck for different mass distributions along head and neck. The neck was assumed to be in a horizontal position. Neck density was assumed as 0.5^−3^. Original, mass distribution as used for the calculations in Figure? 3; heavy head, 20% mass were added to the head and to the foremost section of the neck from c1 to c3; light head, 20% mass was subtracted from the head and from the foremost section of the neck from c1 to c3; light neck base, the base of the neck was assumed to remain elliptical with a width of three quarters of the height instead of becoming circular towards the base of the neck.

The sensitivity analysis with varied neck density (Figure 4, Table 1) and mass distribution along the neck (Figure 5, Table 1) reveals that moderate errors in the estimated mass distribution along the neck do not affect the general result of approximately constant stress values in a horizontal position of the neck, although mass variations of the head and the foremost section of the neck considerably influence the stress along the neck due to the long lever arms of the weight forces at the distal end of the neck.

## Discussion

### Neck posture and feeding strategy

Both the results on vertebral articulation and the results on stress in the intervertebral joint cartilage along the neck support the reconstruction of a nearly straight neck for *Mamenchisaurus youngi*. The orientation of the neck in a habitual posture could have been between slightly declined or inclined up to about 45 degrees. Assuming that the section of the vertebral column behind the second vertebra of the trunk was declining in cranial direction by 10 or 20 degrees (see e.g., Plate II in [24]), the neck in ONP was inclined by about 20 degrees. This result is not very different from the approximately horizontal neck postures that were reconstructed for several sauropods based on the ONP [13], [38].

Recently, it has been questioned whether zygapophyseal alignment yields habitual positions of sauropod necks [27], [40]. Studies on the neck postures of living vertebrates with long necks [26], [27] indicate that the ONP usually is closer to the neck posture during locomotion than to the position of the neck at rest, which is usually by 10 or 20 degrees higher. The comparatively low neck posture during locomotion may be used for increasing forces in epaxial elastic elements along the neck during activity or for shifting forward the center of gravity of the body [26], [27]. Especially in sauropods, a low position of the head during locomotion might be related to a higher metabolic rate compared to standing at rest. With the head well above the heart, an increased blood pressure evokes an additional energy consumption that is proportional to the metabolic rate [20].

In summary, for *Mamenchisaurus youngi*, the results indicate a more or less horizontal, declined, or slightly inclined position of the neck during feeding, a habitual neck posture during locomotion with a slight inclination of about 20 degrees and a habitual neck position during standing at rest with an inclination of approximately 30 or 40 degrees. The pattern of the stress as well as the magnitude of stress values in the intervertebral cartilage along the neck is in accordance with both a horizontal and an inclined position of the neck at rest. Because sauropods would have had a better view over the surrounding area and reduced their vulnerability, it appears reasonable to assume that the neck was kept in an inclined position during standing at rest. The dorsal flexibility at the neck-trunk transition fits this assumption.

A steep inclined or nearly vertical position of the neck is very unlikely even for short time intervals because this would have forced several joints into an extreme position. *Mamenchisaurus youngi*, therefore, probably did not browse at great heights by raising the neck. On the other hand, compared to other neck sections, high ventral flexibility in the midsection of the neck indicates frequent browsing at low heights. In *Diplodocus carnegii* [13], [26], the head could be lowered to ground level by flexion at the base of the neck but also in the midsection of the neck, so that the height of the more massive posterior end of the neck did not change much. Compared to *Diplodocus carnegii*, the overall pattern of dorsoventral flexibility was similar in *Mamenchisaurus youngi*. In contrast to *Diplodocus carnegii*, however, in *Mamenchisaurus youngi* the base of the neck appears to have been rather inclined as opposed to declined, and the neck appears to have been straighter. These features resemble the similarly-sized *Euhelopus zdanskyi* [18], [41], [42]. However, in *Euhelopus zdanskyi*, the vertebral column apparently was flexed more dorsally at the neck-trunk transition than in *Mamenchisaurus youngi* [18], so that the neck possibly was kept in a more inclined position and browsing at great heights cannot be excluded. These findings indicate that *Mamenchisaurus youngi* browsed at lower heights than *Euhelopus zdanskyi*, although the neck mechanics were probably very similar. The comparatively long forelimbs and studies on the intervertebral stress indicate that *Giraffatitan brancai* resembled *Euhelopus* instead of *Mamenchisaurus* in feeding behavior [27]. The posture and utilization of the neck differed more between *Mamenchisaurus youngi* and *Diplodocus carnegii*, though both sauropods may have browsed at low heights. *Mamenchisaurus youngi* may have browsed at medium heights as well.

Like *Euhelopus* and *Giraffatitan* but different from diplodocids, the cervical ribs were very long and overlapping in *Mamenchisaurus*. The evidence recently put forward by Klein et al. [43] supports the hypothesis that cervical ribs were used for transmitting tensile forces along the neck. Yet, the mechanical function of high ventral forces along the neck is not fully clear. Strong tensile structures on the ventral side of the neck might be needed even in a more or less horizontal position of the neck for reducing swinging of the head during locomotion.

The long cervical ribs of *Mamenchisaurus* also support the idea that many sauropod necks had little flexibility. The size, location and orientation of the zygapophyses also indicate little lateral flexibility along the neck. Lateral movements of the neck were more or less restricted to the base of the neck as is frequently found in vertebrates [44]. The wide vertebrae with large, rather flat zygapophyses, starting around the 15^th^ cervical, are well suited for maintaining contact between the pre- and postzygapophyseal joint facets during lateral excursions and for resisting torsion due to sideward movements of the more cranial parts of the neck. In contrast to dorsoventral movements, lateral movements do not imply vertical shifts of the center of mass of the neck. Therefore, it appears reasonable that frequent dorsoventral movements, e.g., during feeding, took place in the more cranial section of the neck (as observed in camels and ostriches (see, e.g., [26]).

### Advantages of a very long neck

The selective advantages of a very long neck, as discussed in the introduction, include increasing access to food, especially for high browsers or reducing energy expenditures, especially in low browsers (e.g., [1], [4], [12], [18], [19]). Simple estimates of energy expenditures have been used to demonstrate advantages of a long neck for different feeding strategies depending on the distribution of food sources [17]–[19].

In addition to increased access to resources and more efficient browsing, a long neck might also have been useful in saving time during feeding intervals. Especially with a patchy distribution of food, with distances between food sources below neck length, the long neck could have served for moving the head quickly from one source to the next. This behavior would not only save energy due to a reduction in body movements and accelerations [17] but would also shorten time intervals between feeding, so that absolute food intake could be increased during a day or during competitive exploration of an area with other herbivores present.

For *Mamenchisaurus youngi*, different selective advantages for a very long neck appear possible. Because of the rather low position and the little flexibility of the neck, it was not useful for exploiting resources at great heights, and it is unlikely that *Mamenchisaurus youngi* walked through dense vegetation. Therefore, it appears reasonable to assume a patchy distribution of food sources. Under this condition, the selective advantage of the long neck might have been to save energy and time by reducing distances that had to be traveled, especially in difficult terrains, or reducing the need to turn or accelerate the whole body. The results may be applied to other mamenchisaurids with similarly constructed necks (e.g., [23]–[25], [45]).

## Conclusions

**Figure 6:**
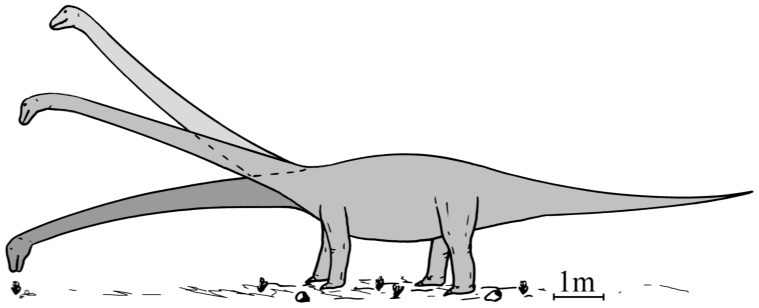
Suggested neck poses for *Mamenchisaurus youngi*. The neck is shown during low browsing, in ONP (middle pose), and in an alert position.

The evidence put forward here indicates that the neck of *Mamenchisaurus youngi* was kept in a more or less straight, not steeply inclined, pose with little mobility in most parts of the neck, as suggested for most sauropods with long necks (e.g., [38], [46]). The functional specialization of the neck sections supports the idea of browsing at low or medium heights: The foremost neck section was comparatively mobile, allowing quick movements over short distances of the head during feeding. Low stress under static conditions in the foremost intervertebral joints indicates muscle activity due to head movements during feeding. The midsection of the neck could be flexed ventrally for low browsing or kept straight or flexed slightly dorsally for browsing at medium heights. The posterior neck section was used for lateral movements of the whole neck, and at the neck-trunk transition, dorsal flexion was performed for raising the neck, e.g. into a resting position. The rather stiff construction of the neck may be related to a low density of vegetation, so that sideward movements of the neck or turning with the whole body were not much restricted by environmental obstacles (see also [15]). During locomotion the neck was slightly inclined. During standing at rest or in an alert position the inclination of the neck could be increased to 30 or 40 degrees (Figure 6).

The results presented here on the neck mechanics and feeding behavior of *Mamenchisaurus youngi*, when compared with the results on other sauropods like *Diplodocus*, *Giraffatitan*, or *Euhelopus*, indicate different ways of using a very long neck among sauropods. Also, there is a considerable variation in body size, dentition and environmental conditions of sauropods (e.g., [47]–[51]) with very long necks, so that niche partitioning among sauropods appears reasonable [47]. Therefore, it has to be concluded that the selective advantage of a long neck was not restricted to the distribution of food, feeding habits, or a very large body size. It appears that multiple advantages made a very long neck stable during the long-term evolution of sauropods [1]. For a greater insight into the selective factors that favored the evolution of very long necks in sauropods, it would be worthwhile to investigate those sauropods that show a reduction in neck length.

## Supporting Information

Table S1 **Dimensions of the zygapophyseal facets at the cervical joints of** ***Mamenchisaurus youngi*** **.** Estimated surface areas of the prezygapophyses (A_pre_) and the postzygapophyses (A_po_) along the neck of *Mamenchisaurus youngi*. The surface areas are calculated from the length and width of the zygapophyseal joint facets as explained in the text. l_pre_, length of the prezygapophyses; w_pre_, width of the prezygapophyses; l_po_, length of the postzygapophyses; w_po_, width of the postzygapophyses. Values are rounded because of deformations of the vertebrae.
(DOC)

Table S2 **Estimated dimensions of neck segments in** ***Mamenchisaurus youngi*** **.** Segment lengths are taken from Table 4 in [24]. Segment heights, segment widths and volumes are estimated as described in the text. For segment volumes, a systematic error up to some ten percent cannot be excluded due to wrong estimates of neck dimensions. This systematic error would be similar for all neck sections. However, a higher error at both, the cranial and caudal ends of the neck are possible because of possible deviations in neck shape in these sections of the neck (see text). Additionally, a statistical error up to 5 percent due to deformations in the vertebrae is possible. Values for volumes are rounded.
(DOC)

Table S3 **Estimated mass distributions along the neck of** ***Mamenchisaurus youngi*** **.** Different estimates of the mass distribution along the head and neck of *Mamenchisaurus youngi* that were used for the calculations of intervertebral stress (Figures 3–5). d 0.5, basic neck reconstruction with a density of 0.5 gcm^−3^ based on the estimated segment volumes given in Table S2; d 0.4, neck with a density of 0.4 gcm^−3^; d 0.6, neck with a density of 0.6 gcm^−3^; d 0.7, neck with a density of 0.7 gcm^−3^; hh, heavy head, neck with a density of 0.5 gcm^−3^ and an increased mass of the head and the foremost section of the neck; lh, light head, neck with a density of 0.5 gcm^−3^ and a reduced mass of the head and the foremost section of the neck; lnb, light neck base, neck with a density of 0.5 gcm^−3^ and a reduced mass of the basal section of the neck. For further explanation see the text.
(DOC)

Table S4 **Mechanically relevant dimensions at the cervical joints of** ***Mamenchisaurus youngi*** **.** The lever arms (h) of epaxial tensile forces and the cross-sectional area (A) of the compressed intervertebral cartilage at the neck joints are estimated as described in the text. a, width of the cotyle; b, height of the cotyle. For a and b rounded values were used at most joints because of slight deformations of the vertebrae. For further explanation see the text.
(DOC)

Table S5 **Dorsoventral flexibility along the neck of** ***Mamenchisaurus youngi*** **.** Ostelogically Neutral Pose (ONP) and maximum possible dorsoventral excursion angles at the intervertebral joints along the neck of *Mamenchisaurus youngi*. Dorsal excursions are positive. The estimated error is 5 degrees for all values. For further explanation see the text.
(DOC)

Table S6 **Stress in intervertebral cartilage along the neck of** ***Mamenchisaurus youngi*** **.** Calculated stress in the intervertebral cartilage along the neck of *Mamenchisaurus youngi* for different hypothetical neck postures and mass distributions along head and neck; b, basic mass reconstruction (with a density of 0.5 gcm^−3^) with a horizontal neck posture; is, basic mass reconstruction with an inclined sigmoidal neck posture; i 45, basic mass reconstruction with a neck inclination of 45 degrees; i 60, basic mass reconstruction with a neck inclination of 60 degrees; de, basic mass reconstruction with a declining neck (−15 degrees); d 0.4, horizontal neck with a density of 0.4 gcm^−3^; d 0.6, horizontal neck with a density of 0.6 gcm^−3^; d 0.7, horizontal neck with a density of 0.7 gcm^−3^; hh (heavy head), horizontal neck with a density of 0.5 gcm^−3^ and an increased mass (20%) of the head and the foremost section of the neck; lh (light head), horizontal neck with a density of 0.5 gcm^−3^ and a reduced mass (20%) of the head and the foremost section of the neck; lnb, light neck base, horizontal neck with a density of 0.5 gcm^−3^ and reduced mass of the basal section of the neck (elliptical shape instead of a transition to a round shape). For further explanation see the text.
(DOC)
