# Supplementary material for: Biomechanical Reconstructions and Selective Advantages of Neck Poses and Feeding Strategies of Sauropods with the Example of Mamenchisaurus youngi
Source: PLoS One. 2013 Oct 30;8(10):e71172. doi: 10.1371/journal.pone.0071172 (PMC3812961; doi:10.1371/journal.pone.0071172)
Supplement: Table S1 — Dimensions of the zygapophyseal facets at the cervical joints of Mamenchisaurus youngi . Estimated surface areas of the prezygapophyses (Apre) and the postzygapophyses (Apo) along the neck of Mamenchisaurus youngi. The surface areas are calculated from the length and width of the zygapophyseal joint facets as explained in the text. lpre, length of the prezygapophyses; wpre, width of the prezygapophyses; lpo, length of the postzygapophyses; wpo, width of the postzygapophyses. Values are rounded because of deformations of the vertebrae. (DOC) [file pone.0071172.s001.doc]

**Table S1. Dimensions of the zygapophyseal facets at the cervical joints of *Mamenchisaurus youngi.***

| Joint | l_pre_ [mm] | w_pre_ [mm] | A_pre_ [mm^2^] | l_po_ [mm] | w_po_ [mm] | A_po_ [mm^2^] |
| --- | --- | --- | --- | --- | --- | --- |
| c2-c3 | 33 | 27 | 700 | - | - | - |
| c3-c4 | - | - | - | - | - | - |
| c4-c5 | 52 | 30 | 1225 | - | - | - |
| c5-c6 | 45 | 38 | 1343 | 40 | 35 | 1100 |
| c6-c7 | - | - | - | 60 | 40 | 1885 |
| c7-c8 | 58 | 35 | 1594 | 55 | 40 | 1728 |
| c8-c9 | 50 | 50 | 1963 | - | - | - |
| c9-c10 | - | - | - | 70 | 50 | 2749 |
| c10-c11 | 60 | 45 | 2121 | - | - | - |
| c11-c12 | 60 | 50 | 2356 | 60 | 60 | 2827 |
| c12-c13 | 75 | 60 | 3534 | 65 | 65 | 3318 |
| c13-c14 | 70 | 60 | 3299 | 65 | 72,5 | 3701 |
| c14-c15 | 55 | - | - | 60 | 80 | 3770 |
| c15-c16 | 65 | 90 | 4595 | 65 | 90 | 4595 |
| c16-c17 | 85 | 110 | 7343 | 90 | 100 | 7069 |
| c17-c18 | 85 | 110 | 7343 | 80 | 110 | 6912 |
| c18-d1 | 80 | 115 | 7226 | 75 | 110 | 6480 |
