# Supplementary material for: Biomechanical Reconstructions and Selective Advantages of Neck Poses and Feeding Strategies of Sauropods with the Example of Mamenchisaurus youngi
Source: PLoS One. 2013 Oct 30;8(10):e71172. doi: 10.1371/journal.pone.0071172 (PMC3812961; doi:10.1371/journal.pone.0071172)
Supplement: Table S2 — Estimated dimensions of neck segments in Mamenchisaurus youngi . Segment lengths are taken from Table 4 in [24]. Segment heights, segment widths and volumes are estimated as described in the text. For segment volumes, a systematic error up to some ten percent cannot be excluded due to wrong estimates of neck dimensions. This systematic error would be similar for all neck sections. However, a higher error at both, the cranial and caudal ends of the neck are possible because of possible deviations in neck shape in these sections of the neck (see text). Additionally, a statistical error up to 5 percent due to deformations in the vertebrae is possible. Values for volumes are rounded. (DOC) [file pone.0071172.s002.doc]

**Table S2. Estimated dimensions of neck segments in *Mamenchisaurus youngi.***

| Segment | Length [m] | Height [m] | Width/Height | Volume [liter] |
| --- | --- | --- | --- | --- |
| neck 1 (c1,c2,c3) | 0.331 | 0.315 | 3/4 | 19.5 |
| neck 2 (c4) | 0.21 | 0.27 | 3/4 | 9 |
| neck 3 (c5) | 0.26 | 0.28 | 3/4 | 12 |
| neck 4 (c6) | 0.32 | 0.295 | 3/4 | 16.5 |
| neck 5 (c7) | 0.37 | 0.31 | 3/4 | 21 |
| neck 6 (c8) | 0.405 | 0.33 | 3/4 | 26 |
| neck 7 (c9) | 0.417 | 0.35 | 3/4 | 30 |
| neck 8 (c10) | 0.44 | 0.37 | 3/4 | 35.5 |
| neck 9 (c11) | 0.455 | 0.395 | 3/4 | 42 |
| neck 10 (c12) | 0.46 | 0.42 | 3/4 | 48 |
| neck 11 (c13) | 0.45 | 0.455 | 3/4 | 55 |
| neck 12 (c14) | 0.49 | 0.49 | 3/4 | 69 |
| neck 13(c15) | 0.41 | 0.53 | 3/4 · 1.1 | 75 |
| neck 14 (c16) | 0.365 | 0.57 | 3/4 · 1.2 | 84 |
| neck 15 (c17) | 0.316 | 0.62 | 3/4 · 1.3 | 93 |
| neck 16 (c18) | 0.26 | 0.69 | 1 | 97 |
| neck total | 5.959 |  |  | 732.5 |
