# Supplementary material for: Biomechanical Reconstructions and Selective Advantages of Neck Poses and Feeding Strategies of Sauropods with the Example of Mamenchisaurus youngi
Source: PLoS One. 2013 Oct 30;8(10):e71172. doi: 10.1371/journal.pone.0071172 (PMC3812961; doi:10.1371/journal.pone.0071172)
Supplement: Table S3 — Estimated mass distributions along the neck of Mamenchisaurus youngi . Different estimates of the mass distribution along the head and neck of Mamenchisaurus youngi that were used for the calculations of intervertebral stress (Figures 3– 5). d 0.5, basic neck reconstruction with a density of 0.5 gcm−3 based on the estimated segment volumes given in Table S2; d 0.4, neck with a density of 0.4 gcm−3; d 0.6, neck with a density of 0.6 gcm−3; d 0.7, neck with a density of 0.7 gcm−3; hh, heavy head, neck with a density of 0.5 gcm−3 and an increased mass of the head and the foremost section of the neck; lh, light head, neck with a density of 0.5 gcm−3 and a reduced mass of the head and the foremost section of the neck; lnb, light neck base, neck with a density of 0.5 gcm−3 and a reduced mass of the basal section of the neck. For further explanation see the text. (DOC) [file pone.0071172.s003.doc]

**Table S3. Estimated mass distributions along the neck of *Mamenchisaurus youngi.***

|  | Mass [kg] | | | | | | |
| --- | --- | --- | --- | --- | --- | --- | --- |
| Segment | d 0.5 | d 0.4 | d 0.6 | d 0.7 | hh | lh | lnb |
| head | 25 | 25 | 25 | 25 | 30 | 20 | 25 |
| neck 1 (c1,c2,c3) | 9.75 | 7.8 | 11.7 | 13.65 | 11.7 | 7.8 | 9.75 |
| neck 2 (c4) | 4.5 | 3.6 | 5.4 | 6.3 | 4.5 | 4.5 | 4.5 |
| neck 3 (c5) | 6 | 4.8 | 7.2 | 8.4 | 6 | 6 | 6 |
| neck 4 (c6) | 8.25 | 6.6 | 9.9 | 11.55 | 8.25 | 8.25 | 8.25 |
| neck 5 (c7) | 10.5 | 8.4 | 12.6 | 14.7 | 10.5 | 10.5 | 10.5 |
| neck 6 (c8) | 13 | 10.4 | 15.6 | 18.2 | 13 | 13 | 13 |
| neck 7 (c9) | 15 | 12 | 18 | 21 | 15 | 15 | 15 |
| neck 8 (c10) | 17.75 | 14.2 | 21.3 | 24.85 | 17.75 | 17.75 | 17.75 |
| neck 9 (c11) | 21 | 16.8 | 25.2 | 29.4 | 21 | 21 | 21 |
| neck 10 (c12) | 24 | 19.2 | 28.8 | 33.6 | 24 | 24 | 24 |
| neck 11 (c13) | 27.5 | 22 | 33 | 38.5 | 27.5 | 27.5 | 27.5 |
| neck 12 (c14) | 34.5 | 27.6 | 41.4 | 48.3 | 34.5 | 34.5 | 34.5 |
| neck 13(c15) | 37.5 | 30 | 45 | 52.5 | 37.5 | 37.5 | 34.09 |
| neck 14 (c16) | 42 | 33.6 | 50.4 | 58.8 | 42 | 42 | 35 |
| neck 15 (c17) | 46.5 | 37.2 | 55.8 | 65.1 | 46.5 | 46.5 | 35.77 |
| neck 16 (c18) | 48.5 | 38.8 | 58.2 | 67.9 | 48.5 | 48.5 | 36.38 |
| total | 391.25 | 318 | 464.5 | 537.75 | 398.2 | 384,3 | 357,99 |
