# Supplementary material for: Biomechanical Reconstructions and Selective Advantages of Neck Poses and Feeding Strategies of Sauropods with the Example of Mamenchisaurus youngi
Source: PLoS One. 2013 Oct 30;8(10):e71172. doi: 10.1371/journal.pone.0071172 (PMC3812961; doi:10.1371/journal.pone.0071172)
Supplement: Table S4 — Mechanically relevant dimensions at the cervical joints of Mamenchisaurus youngi . The lever arms (h) of epaxial tensile forces and the cross-sectional area (A) of the compressed intervertebral cartilage at the neck joints are estimated as described in the text. a, width of the cotyle; b, height of the cotyle. For a and b rounded values were used at most joints because of slight deformations of the vertebrae. For further explanation see the text. (DOC) [file pone.0071172.s004.doc]

**Table S4. Mechanically relevant dimensions at the cervical joints of *Mamenchisaurus youngi.***

| Joint | h [mm] | a [mm] | b [mm] | A [mm^2^] |
| --- | --- | --- | --- | --- |
| c3-c4 | 95 | 62 | 72 | 3506 |
| c4-c5 | 110 | 56(?) | 70(?) | (3079) |
| c5-c6 | 120 | 66 | 65 | 3369 |
| c6-c7 | 130 | 75 | 80 | 4712 |
| c7-c8 | 145 | 89 | 93 | 6501 |
| c8-c9 | 165 | 95 | 100 | 7461 |
| c9-c10 | 190 | 96 | 110 | 8294 |
| c10-c11 | 215 | 105 | 128 | 10556 |
| c11-c12 | 245 | 115 | 127 | 11471 |
| c12-c13 | 275 | 120 | 150 | 14137 |
| c13-c14 | 300 | 130 | 160 | 16336 |
| c14-c15 | 320 | 125 | 185 | 18162 |
| c15-c16 | 330 | 140 | 180 | 19792 |
| c16-c17 | 330? | 150 | 185 | 21795 |
| c17-c18 | 330 | 145 | 210 | 23915 |
| c18-d1 | 335 | 140 | 210 | 23090 |
