# Supplementary material for: Biomechanical Reconstructions and Selective Advantages of Neck Poses and Feeding Strategies of Sauropods with the Example of Mamenchisaurus youngi
Source: PLoS One. 2013 Oct 30;8(10):e71172. doi: 10.1371/journal.pone.0071172 (PMC3812961; doi:10.1371/journal.pone.0071172)
Supplement: Table S5 — Dorsoventral flexibility along the neck of Mamenchisaurus youngi . Ostelogically Neutral Pose (ONP) and maximum possible dorsoventral excursion angles at the intervertebral joints along the neck of Mamenchisaurus youngi. Dorsal excursions are positive. The estimated error is 5 degrees for all values. For further explanation see the text. (DOC) [file pone.0071172.s005.doc]

**Table S5. Dorsoventral flexibility along the neck of *Mamenchisaurus youngi.***

| Joint | ONP [degree] | Dorsal [degree] | Ventral [degree] |
| --- | --- | --- | --- |
| c3-c4 | -17.5 | 10 | -40 |
| c4-c5 | -7.5 | 27.5 | -22.5 |
| c5-c6 | -5 | 20 | -25 |
| c6-c7 | 0 | 25 | -20 |
| c7-c8 | -5 | 20 | -20 |
| c8-c9 | 0 | 20 | -20 |
| c9-c10 | -5 | 15 | -20 |
| c10-c11 | 0 | 15 | -25 |
| c11-c12 | 0 | 15 | -25 |
| c12-c13 | 0 | 12.5 | -25 |
| c13-c14 | 0 | 10 | -20 |
| c14-c15 | 0 | 15 | -20 |
| c15-c16 | 0 | 20 | -20 |
| c16-c17 | 5 | 20 | -15 |
| c17-c18 | 10 | 22.5 | -5 |
| c18-d1 | 12.5 | 22.5 | -2.5 |
| d1-d2 | 12.5 | 20 | -2.5 |
