# Supplementary material for: Biomechanical Reconstructions and Selective Advantages of Neck Poses and Feeding Strategies of Sauropods with the Example of Mamenchisaurus youngi
Source: PLoS One. 2013 Oct 30;8(10):e71172. doi: 10.1371/journal.pone.0071172 (PMC3812961; doi:10.1371/journal.pone.0071172)
Supplement: Table S6 — Stress in intervertebral cartilage along the neck of Mamenchisaurus youngi . Calculated stress in the intervertebral cartilage along the neck of Mamenchisaurus youngi for different hypothetical neck postures and mass distributions along head and neck; b, basic mass reconstruction (with a density of 0.5 gcm−3) with a horizontal neck posture; is, basic mass reconstruction with an inclined sigmoidal neck posture; i 45, basic mass reconstruction with a neck inclination of 45 degrees; i 60, basic mass reconstruction with a neck inclination of 60 degrees; de, basic mass reconstruction with a declining neck (−15 degrees); d 0.4, horizontal neck with a density of 0.4 gcm−3; d 0.6, horizontal neck with a density of 0.6 gcm−3; d 0.7, horizontal neck with a density of 0.7 gcm−3; hh (heavy head), horizontal neck with a density of 0.5 gcm−3 and an increased mass (20%) of the head and the foremost section of the neck; lh (light head), horizontal neck with a density of 0.5 gcm−3 and a reduced mass (20%) of the head and the foremost section of the neck; lnb, light neck base, horizontal neck with a density of 0.5 gcm−3 and reduced mass of the basal section of the neck (elliptical shape instead of a transition to a round shape). For further explanation see the text. (DOC) [file pone.0071172.s006.doc]

**Table S6. Stress in intervertebral cartilage along the neck of *Mamenchisaurus youngi.***

|  | Stress [kPa] | | | | | | | | | | |
| --- | --- | --- | --- | --- | --- | --- | --- | --- | --- | --- | --- |
| Joint | b | is | i 45 | i 60 | de | d 0.4 | d 0.6 | d 0.7 | hh | lh | lnb |
| c3-c4 | 456 | 484 | 436 | 390 | 422 | 446 | 466 | 476 | 547 | 365 | 456 |
| c4-c5 | 610 | 631 | 550 | 470 | 566 | 588 | 632 | 654 | 729 | 490 | 610 |
| c5-c6 | 831 | 825 | 714 | 590 | 775 | 788 | 873 | 915 | 985 | 676 | 831 |
| c6-c7 | 801 | 762 | 666 | 535 | 750 | 748 | 854 | 907 | 939 | 664 | 801 |
| c7-c8 | 747 | 667 | 608 | 480 | 700 | 687 | 807 | 868 | 863 | 631 | 747 |
| c8-c9 | 799 | 652 | 644 | 502 | 750 | 724 | 875 | 951 | 911 | 688 | 799 |
| c9-c10 | 844 | 624 | 678 | 525 | 790 | 754 | 934 | 1,024 | 949 | 739 | 844 |
| c10-c11 | 778 | 557 | 623 | 481 | 728 | 687 | 869 | 960 | 864 | 692 | 778 |
| c11-c12 | 819 | 587 | 657 | 508 | 765 | 715 | 923 | 1,027 | 900 | 739 | 819 |
| c12-c13 | 758 | 553 | 609 | 471 | 707 | 655 | 860 | 963 | 824 | 692 | 758 |
| c13-c14 | 753 | 563 | 606 | 468 | 702 | 646 | 860 | 968 | 812 | 694 | 753 |
| c14-c15 | 800 | 615 | 644 | 497 | 746 | 681 | 919 | 1,038 | 855 | 745 | 800 |
| c15-c16 | 857 | 669 | 689 | 531 | 798 | 725 | 989 | 1,121 | 910 | 803 | 856 |
| c16-c17 | 915 | 710 | 734 | 565 | 853 | 770 | 1.060 | 1,205 | 967 | 863 | 911 |
| c17-c18 | 960 | 739 | 769 | 592 | 895 | 804 | 1.115 | 1,270 | 1,010 | 909 | 949 |
| c18-d1 | 1,100 | 857 | 884 | 682 | 1,024 | 919 | 1,281 | 1,462 | 1,153 | 1,046 | 1,080 |
|  |  |  |  |  |  |  |  |  |  |  |  |
